# Air Pollution in China: Mapping of Concentrations and Sources

**DOI:** 10.1371/journal.pone.0135749

**Published:** 2015-08-20

**Authors:** Robert A. Rohde, Richard A. Muller

**Affiliations:** 1 Berkeley Earth, Berkeley, California, United States of America; 2 Department of Physics, University of California, Berkeley, California, United States of America; Nanjing University, CHINA

## Abstract

China has recently made available hourly air pollution data from over 1500 sites, including airborne particulate matter (PM), SO_2_, NO_2_, and O_3_. We apply Kriging interpolation to four months of data to derive pollution maps for eastern China. Consistent with prior findings, the greatest pollution occurs in the east, but significant levels are widespread across northern and central China and are not limited to major cities or geologic basins. Sources of pollution are widespread, but are particularly intense in a northeast corridor that extends from near Shanghai to north of Beijing. During our analysis period, 92% of the population of China experienced >120 hours of unhealthy air (US EPA standard), and 38% experienced average concentrations that were unhealthy. China’s population-weighted average exposure to PM_2.5_ was 52 μg/m^3^. The observed air pollution is calculated to contribute to 1.6 million deaths/year in China [0.7–2.2 million deaths/year at 95% confidence], roughly 17% of all deaths in China.

## Introduction

Air pollution is a problem for much of the developing world and is believed to kill more people worldwide than AIDS, malaria, breast cancer, or tuberculosis [1–4]. Airborne particulate matter (PM) is especially detrimental to health [5–8], and has previously been estimated to cause between 3 and 7 million deaths every year, primarily by creating or worsening cardiorespiratory disease [2–4,6,7]. Particulate sources include electric power plants, industrial facilities, automobiles, biomass burning, and fossil fuels used in homes and factories for heating. In China, air pollution was previously estimated to contribute to 1.2 to 2 million deaths annually [2–4].

In 2012, China adopted the Ambient Air Quality Standard [9], and began development of a national Air Reporting System that now includes 945 sites in 190 cities. These automated stations report hourly via the internet, and focus on six pollutants: particulate matter < 2.5 microns (PM_2.5_), particulate matter < 10 microns (PM_10_), sulfur dioxide (SO_2_), nitrogen dioxide (NO_2_), ozone (O_3_), and carbon monoxide (CO). Provincial governments perform air quality monitoring at 600 additional locations that are not yet integrated into the national system. Previous studies of regional scale air pollution have generally relied on satellite data [10,11] or modeling [12,13], but the high density of hourly data in China now allows regional patterns to be constructed directly from ground observations.

## Materials and Methods

Though China deserves praise for its monitoring system and transparent communication, most archived observations are not publicly available. To compensate, real-time data was downloaded every hour during a four month interval from April 5, 2014 to August 5, 2014. Due to download restrictions on the official Chinese air quality reporting system, two different third-party sources were used: PM25.in and AQICN.org. PM25.in is a direct mirror of data from the 945 stations in China’s national network, while AQICN.org is the world’s largest aggregator of real-time air quality data and included many additional sites in China and surrounding areas. Nearly all of the additional data from within China originates from stations operated by provincial environmental agencies that have not yet been incorporated in China’s national network.

Consistency, quality control, and validation checks were applied to the raw data prior to further analysis in order to reduce the impact of outliers, badly calibrated instruments, and other problems. The most common quality problem was associated with stuck instruments that implausibly reported the same concentration continuously for many hours. A regional consistency check was also applied to verify that each station was reporting data similar to its neighboring stations. Approximately 8% of the data was removed as a result of the quality control review. Further details are described in the supplemental material (S1 Text).

**Fig 1:**
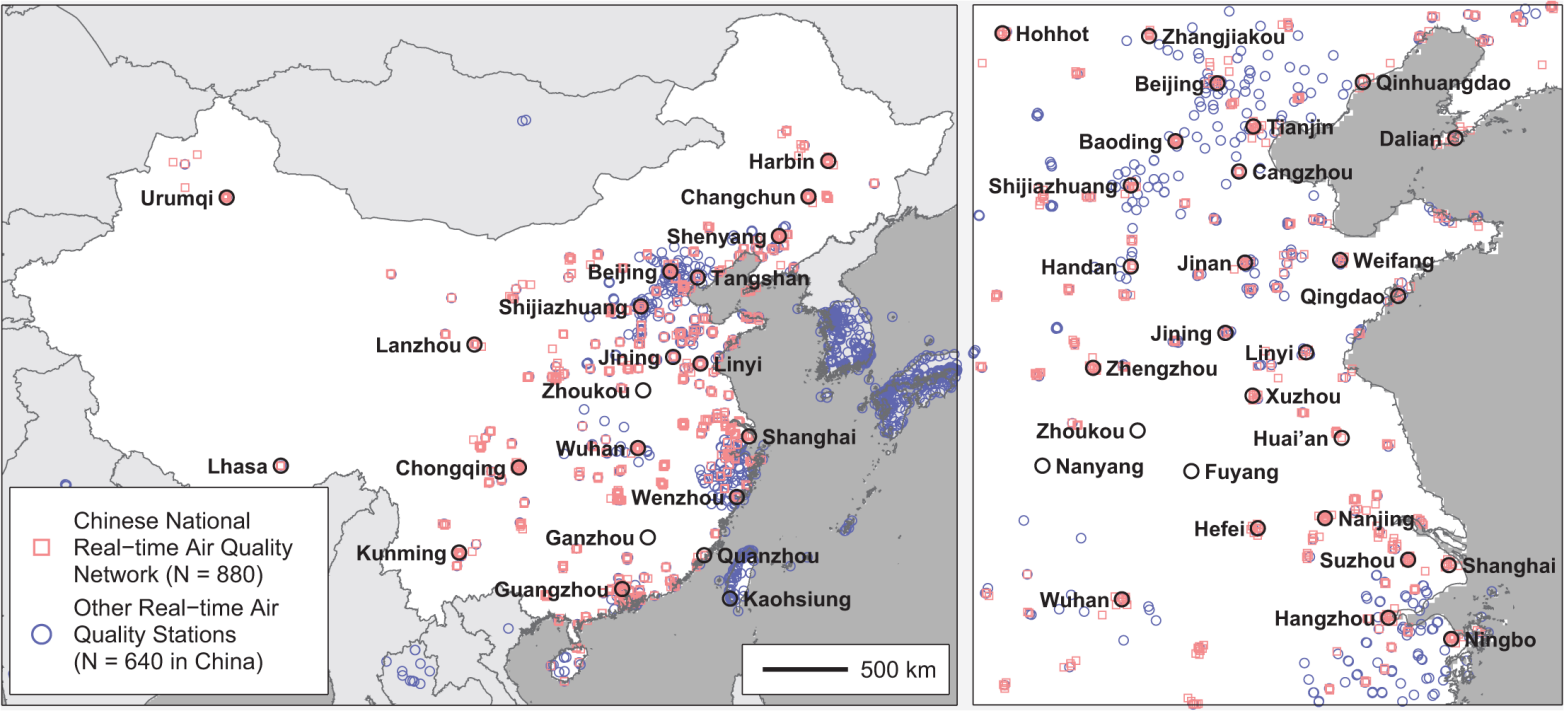
Map of real-time air pollution monitoring stations. Map shows the locations of air quality monitoring sites in China and surrounding areas with sufficient hourly data to be included in this study. Selection criteria and data sources are described in S1 Text. The map was prepared in MATLAB using political boundaries from the Global Database of Administrative Areas (version 2; http://gadm.org/).

As little monitoring is conducted in western China (Fig 1); we will focus on China east of 95° E, which includes 97% of the population. After removing stations with a high percentage of missing values or with other quality control problems, this study used 880 national network sites, 640 other sites in China and Taiwan, and 236 sites in other countries within 500 km of China (mostly South Korea). The air quality network is skewed towards urban areas, often with several sites per city and fewer, if any, in rural areas. For the *n*-th site, we use *p*_*n*_(*t*) to denote the pollutant concentration time series and $\bar{p_{n}}$ to denote the mean pollutant concentration.

For each pollutant, a correlation vs. distance function was estimated by computing all possible pairwise correlations between different stations and fitting the resulting correlations to a two part exponential decay as a function of distance. The resulting functional forms are stated in S2 Table and shown in S2 and S3 Figs. The correlation functions are used to construct correlation matrices that in turn are used to compute Kriging coefficients [14, 15], $K_{n}\left(\vec{x}\right)$.

The interpolated pollutant field, $P\left(\vec{x},t\right)$, is then estimated in two parts.

$$P\left(\vec{x},t\right)=S\left(\vec{x}\right)+A\left(\vec{x},t\right)$$

$$S\left(\vec{x}\right)=\left(\sum_{n}K_{n}\left(\vec{x}\right)\left(\bar{p_{n}}-G\left({\vec{x}}_{n}\right)\right)\right)+G\left(\vec{x}\right)$$

$$A\left(\vec{x},t\right)=\sum_{n}K_{n}^{*}\left(\vec{x},t\right)\left(p_{n}\left(t\right)-\bar{p_{n}}\right)$$

The stationary part, $S\left(\vec{x}\right)$, is derived by applying Kriging interpolation to the mean pollutant concentrations and a global predictor, $G\left({\vec{x}}_{n}\right)$, that depends on latitude and longitude and contains free parameters that are adjusted to fit the observed means. The time-dependent anomaly part, $A\left(\vec{x},t\right)$, depends only on the fluctuations at each station relative to the local mean, and its Kriging coefficients, $K_{n}^{*}\left({\vec{x}}_{n},t\right)$, are computed with restriction to stations that are active at time *t*. This two-step process reduces errors associated with stations that have intermittently missing data. This method is similar to that used by Berkeley Earth for its historical earth temperature analysis [16]. Since the correlation vs. distance function has been constructed with the correlation at zero distance obtaining a value less than one, the resulting interpolated fields will be smoother than the original data. This design was chosen for its ability to compensate for noise in the underlying station measurements. Additional details of the interpolation process are provided in the supplement methods (S1 Text).

For mapping and computation, this continuous field was sampled with an approximately 6 km resolution, though in practice, the characteristic size of resolvable features is often larger (e.g. 30 km) and varies with station density and noise.

A simple estimate of pollutant fluxes, $F\left(\vec{x},t\right)$, was computed by comparing observed changes in the hourly pollutant concentration to the concentrations expected due to short-term wind transport $\vec{v}\left(\vec{x},t\right)$ and an exponential decay with lifetime *τ*. Differences from the simple transport and decay model are assumed to represent source fluxes.

$$F\left(\vec{x},t\right)=\frac{P\left(\vec{x}+\vec{v}\left(\vec{x},t\right)\;\Delta t,t+\Delta t\right)-e^{-\Delta t/\tau }P\left(\vec{x},t\right)}{2\Delta t}+\frac{P\left(\vec{x},t\right)-e^{-\Delta t/\tau }P\left(\vec{x}-\vec{v}\left(\vec{x},t\right)\;\Delta t,t-\Delta t\right)}{2\Delta t}$$

The near-surface (80 m) wind field from the Global Forecast System [17] was used for this calculation, and the effective pollutant lifetime was estimated as described in S1 Text and reported in S2 Table. Flux averages were computed by time-averaging the resulting field after excluding outlying values and cells affected by rain events as determined from Tropical Rainfall Measuring Mission data [18, 19].

The change in mortality due to PM_2.5_ air pollution was calculated by adopting the integrated exposure response function approach [20] which considers relative risk of death for five disease classes (stroke, ischemic heart disease, lung cancer, chronic obstructive pulmonary disease, and lower respiratory infection) and which was adopted by World Health Organization (WHO) for the Global Burden of Disease study [21]. The model incorporates non-linear response versus concentration and provides an estimate of uncertainty. Relative risk was calculated at the prefecture level using local average PM_2.5_ concentration. The data for different diseases and prefectures was then combined to construct national average mortality estimates.

Analysis and figure rendering was performed using original software written for this project on the MATLAB platform (version 2014a; http://www.mathworks.com/). Additional details of these calculations and associated background information is provided in the supplemental methods document (S1 Text).

## Results

**Fig 2:**
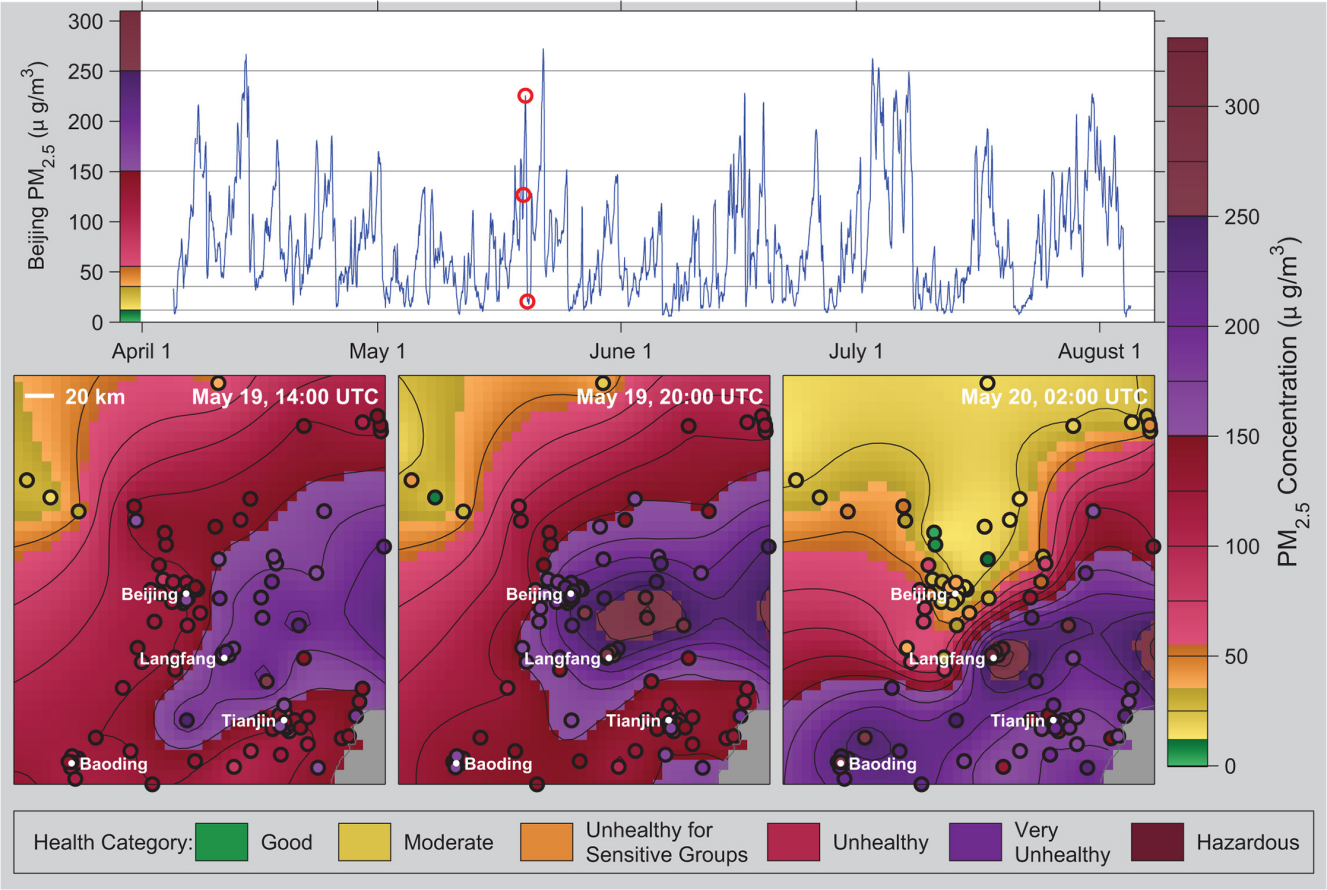
Time evolution of PM_2.5_ pollution in the vicinity of Beijing. (Top) Time series of PM_2.5_ concentration at Beijing extracted from the interpolated field. Red circles indicate times shown in bottom row. (Bottom) Maps of interpolated PM_2.5_ concentration during a period of high pollution. Pollution concentrations were computed as described in the text from hourly data and maps were rendered in MATLAB. Concentrations are shown using color gradients and contour lines, where color tones (green, yellow, etc.) correspond to health impact categories defined by the US EPA. Bold circles show station locations with the observed value at each station indicated by the color within the circle.

Fig 2 shows a time series of PM_2.5_ concentration at Beijing and interpolated maps at three time points separated by 6 hours each. This shows the volatile nature of air pollution and the role of weather patterns in redistributing pollution on short timescales. Our approach creates a smooth field that approximates the data at each station, but allows a degree of difference attributable to noise. The pollution is extensive and rapidly evolves in response to winds and other atmospheric conditions. In the figure, fresh air from the North displaces a period of heavy pollution. Hourly data allows us to capture this evolution and ultimately estimate source fluxes. S1 Movie shows the time evolution of PM_2.5_ across the entire country.

**Fig 3:**
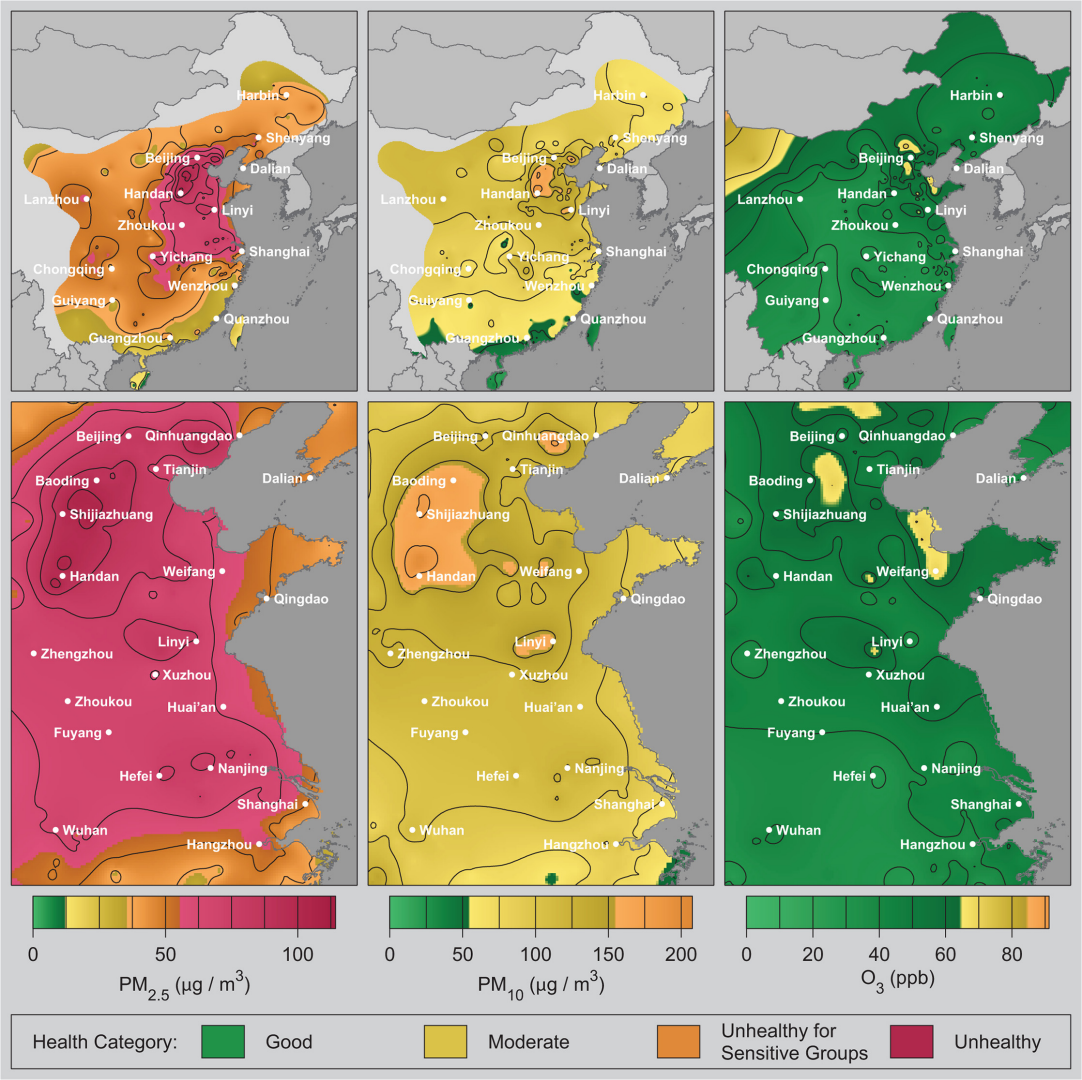
Average air pollution maps. Maps of average pollutant concentration for PM_2.5_, PM_10_, and O_3_ for eastern China (top row) and the Beijing to Shanghai corridor (bottom row). Concentrations are shown using color gradients and contour lines; the colors (green, yellow, etc.) represent US EPA qualitative health impacts. Pollution concentrations were computed as described in the text using hourly data and then the hourly concentration fields were averaged over the four month study duration. The resulting maps were rendered using MATLAB.

Fig 3 presents averages of the interpolated data for PM_2.5_, PM_10_, and O_3_ across the study duration. The maps are color-coded based on US EPA health categories for 24-hour exposure [22]. Maps for SO_2_ and NO_2_ are included in the supplemental materials and show “good” levels nearly everywhere (S11 and S13 Figs).

Air pollution is extensive in China, with the highest particulate concentrations observed south of Beijing (e.g. Xingtai / Handan), but significant levels extend throughout the interior, which is consistent with previous satellite and modeling estimates [11–13]. Extensive pollution is not surprising since particulate matter can remain airborne for days to weeks and travel thousands of kilometers. The corridor south of Beijing contains the highest pollution concentrations and, as discussed below, many of the largest sources. During this study, the southern coastal area experienced somewhat better air quality, possibly linked to greater rainfall (S1 Fig).

For PM_2.5_, portions of China encompassing roughly 38% of the population are classified as “unhealthy” on average (>55 μg/m^3^, red) with an additional 45% of the population averaging “unhealthy for sensitive groups” (>35 μg/m^3^, orange). Almost none of the study area averaged below the US EPA’s 12 μg/m^3^ standard for annual average PM_2.5_ exposure (green). The area-weighted average was 46 μg/m^3^ and the population-weighted average exposure to PM_2.5_ was 52 μg/m^3^. 92% of China’s population experienced unhealthy PM_2.5_ for at least 120 hours during the study period. 46% of China’s population experienced PM_2.5_ above the highest EPA threshold (“hazardous”, >250 μg/m^3^), during at least one hour in the observation period.

Patterns for PM_10_ are similar but less severe, with average PM_10_ levels “moderate” for most of China. Ozone concentrations are modest across most of China, though higher levels occur in the Northwest desert area, and in a small number of Northeastern cities. Though the average levels of PM_10_ and O_3_ are “moderate” or “good” for much of China, intermittently high levels of these pollutants can occur in some areas.

### Source Regions for Air Pollution in China

**Fig 4:**
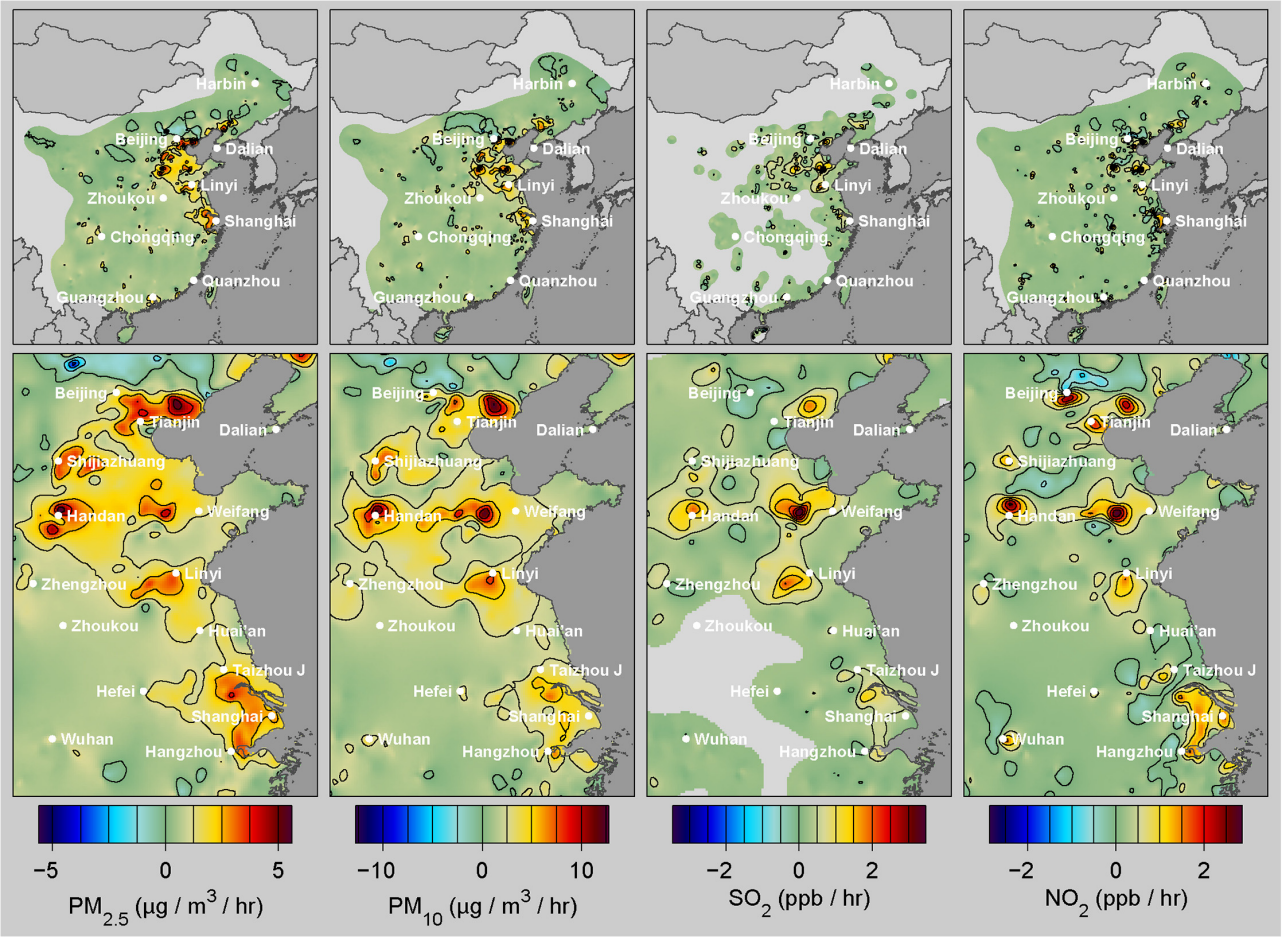
Air pollution source maps. Maps of average pollutant flux for PM_2.5_, PM_10_, SO_2_, and NO_2_ for eastern China (top row) and the Beijing to Shanghai corridor (bottom row). Pollutant fluxes were computed as described in the text from changes in the interpolated hourly pollution fields along with contemporaneous wind and weather data. Due to sparse sampling and secondary transformations of pollutants in the atmosphere, apparent source fluxes are likely to appear more diffuse than the true emissions source.

Fig 4 shows estimated pollutant fluxes for PM_2.5_, PM_10_, SO_2_, and NO_2_. Pollution emission is often localized, especially in the Beijing to Shanghai corridor where many of the highest PM concentrations also occur (Fig 3). Most of the largest emissions appear in or near urban areas (e.g. Handan, Shijazhuang, Zibo, Tangshan, Linyi, Hangshou), though not all major cities have high pollution fluxes (e.g. Chongqing, Chengdu, Wuhan). The source map presumably reflects patterns of industrial activity, though detailed differences will not be explored here.

NO_2_ and SO_2_ emissions help suggest the pollution source. Nitrogen oxides, including nitric oxide (NO) and NO_2_ are created when air is heated, and on average have been attributed to transportation fuels (15–25%), fossil fuel burning in power plants (30–50%), and to industrial facilities (25–35%) [23, 24]. It is expected that NO dominates at the combustion source, but in the presence of sunlight NO and NO_2_ will equilibrate within a few minutes (as well as reacting with O_3_), implying that NO_2_ measurements reflect a combination of NO and NO_2_ emissions. SO_2_ emissions have been previously associated with coal (~90%) in power plants and industrial facilities [25]. Beijing has negligible SO_2_ flux, despite a large NO_2_ signal, possibly a result of policies that limit coal burning in the immediate vicinity of Beijing and more extensively apply mitigation technologies.

Many of the SO_2_ and NO_2_ sources are also sources of PM pollution. This is not surprising since fossil fuel burning is also a major source of PM_2.5_ and PM_10_. However, the PM sources appear more diffuse than either the SO_2_ or NO_2_ sources. In part, this is caused by secondary particulate matter formed within the atmosphere from other pollutants, such as SO_2_ or NO_2_ [26]. Secondary particulate formation may cause PM fluxes to appear more widely distributed than the underlying emitters. Nonetheless, many strong PM sources are identified through this analysis. Within the study region, 10% of the area is responsible for 34% of the PM_2.5_ emissions, and 5% of the area is responsible for 22% of emissions. However, small and moderate sources are also important. Approximately 37% of the study region had PM_2.5_ fluxes >0.5 μg/m^3^/hr, sufficient to exceed US EPA standards after only 3 days of stagnant air.

## Discussion

We have presented a technique for mapping air pollution concentrations and sources using data from monitoring stations. As has been known from satellite and modeling studies, particulate pollution is an extensive problem affecting nearly all of China’s population, but the observed heterogeneity of source locations could help develop strategies to reduce pollution.

We examined a four month interval as long-term station data were not available for most of China. Previous studies of both in situ and satellite data have indicated that winter and early spring months in China have somewhat higher PM concentrations due to increased use of fossil fuels for seasonal heating, weather patterns that concentrate pollution at low altitudes, and increased desert dust fluxes [12, 27]. In contrast, the air in China is typically cleanest from late summer to early fall. The April 5 to August 5 study period is somewhat intermediate. A review of hourly PM_2.5_ station data from Beijing (2009–2014), Shanghai (2012–2014), Guangzhou (2012–2014), Chengdu (2013–2014), and Shenyang (2014) indicates that the months studied in this paper averaged 91%, 84%, 89%, 72%, and 73% respectively of the annual averages (U.S. Air Quality Monitoring Program, http://www.stateair.net/web/mission/1/). Monthly-resolved PM_2.5_ satellite data for the whole study region was not immediately available, but a monthly satellite history for Beijing reported that April-July averaged 99% of the annual mean during 2000 to 2012 [28]. Hence, particulate pollution estimates drawn from the current short study period will likely be similar to or somewhat lower than long-term averages. Future work could explore seasonal variations and long-term trends.

**Fig 5:**
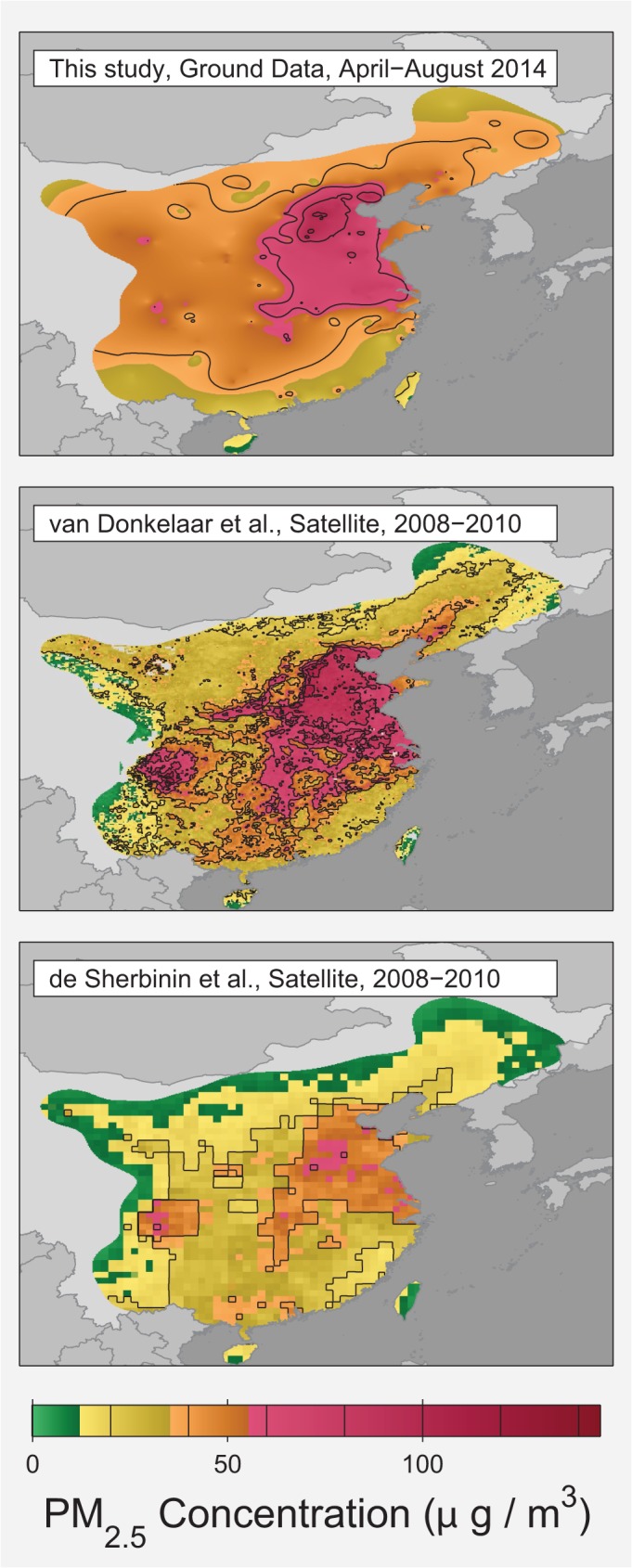
Comparison of PM_2.5_ observations to satellite data. Maps of average PM_2.5_ concentration from this study (top) and two satellite-derived datasets restricted to the same region. The average over the 2008 to 2010 time interval was chosen for the satellite data due to the limitations of the available satellite data. Both the concentrations reported by van Donkelaar [28] (middle) and those reported by de Sherbinin [30] (bottom) rely on similar satellite observations of aerosol optical depth (obtained by NASA), but interpret those observations differently when determining pollutant concentration. The satellite-derived data was imported from concentration data files provided by their respected sources and rendered via MATLAB to use the same US EPA health category color scheme applied in Figs 2 and 3.

During the four months studied, the population-weighted and area-weighted PM_2.5_ averages were 52 and 46 μg/m^3^ respectively. Satellite pollution datasets generally focus on annual or multi-year averages, which limits the ability to make direct comparisons. However, the available satellite estimates tend to be similar to or somewhat lower than the ground observations. An analysis of the larger East Asia region estimated a population-weighted PM_2.5_ exposure of 50 μg/m^3^ for 2001 to 2010 [28]. A version of the same dataset masked to the current study region had an area-weighted average of 40 μg/m^3^ from 2010 to 2012 [28, 29]. A different satellite estimate using similar observations but different calibrations and modeling gave 25 μg/m^3^ for the 2008 to 2010 average over the present study region [30, 31]. Both of these datasets show similar spatial patterns to what we observe (Fig 5), though the magnitudes in van Donkelaar et al.’s work [28] are clearly more consistent with our ground data estimates. As noted in previous satellite to in situ comparisons, satellite data may be more likely to underestimate pollution concentrations during the most extreme pollution events [28, 32].

The conversion of pollution concentrations to mortality is complicated. We adopt the framework [20] developed for the WHO Global Burden of Disease study [21] that considers PM_2.5_ mortality due to impacts on five distinct diseases and accounts for nonlinearities as a function of concentration. Using prefecture level population and pollution data along with national average death rates for the five modeled diseases, we calculate that 1.6 million deaths / year can be attributed to PM_2.5_ air pollution under the WHO model [95% confidence: 0.7 to 2.2 million deaths/year]. This is equivalent to 4 thousand deaths / day or 17% of all deaths in China. Additional details appear in S1 Text and S1 Table. For perspective, the categories of mortality events considered by the WHO model, e.g. cardiorespiratory deaths, account for roughly 55% of all Chinese deaths [21]. This compares to only 42% of mortality in the United States in the same cardiorespiratory categories, despite much higher incidence of obesity in the United States [21]. The calculated mortality is somewhat higher than the 1.2 million deaths/year previously estimated from the Huai River study using Chinese air pollution measurements and mortality data [3,4,33].

Though most of China is subject to potentially harmful levels of PM_2.5_, some large population centers (Chongqing, Wuhan, Chengdu) emit less than half the PM_2.5_ of others. Among northeastern cities, Beijing has relatively low emissions except for NO_2_. Low SO_2_ fluxes may indicate cities that benefit from lower coal usage or better smokestack pollution controls. Compared to natural gas, coal produces 150 to 400 times more PM for the same energy delivered [34,35]. China has plans for new coal plants in the next decade that could effectively double their coal consumption [36], potentially exacerbating the problem of air pollution. A table of pollution concentrations and fluxes by province and prefecture is included in S1 Table.

The methods of this study should be applicable to air quality monitoring in other regions of the world. However, these techniques require an extensive air-quality monitoring network with frequent updates (e.g. hourly), and such networks presently exist in only a few places. We hope that other countries will follow China’s lead and provide both extensive and transparent real-time air quality monitoring.

## Supporting Information

S1 Text Supplemental Methods. Additional discussion of methods, data handling, and validation.
(DOCX)

S1 Table Province and prefecture pollution and mortality data. Provides pollution concentration and flux data broken down by geographic regions within China. Also provides the details of the associated mortality calculation.
(XLSX)

S2 Table Correlation vs. distance and lifetime of air pollutants in China. Summary of the empirically determined correlation vs. distance functions for the five pollutants discussed in this study and the effective residence time in the atmosphere of their pollutant plumes in the absence of rain or snow. The correlation functions generally consist of a short range component (influenced largely by source distributions) and a long-range component (influenced by weather patterns). The effective pollutant plume lifetime shows the value empirically estimated for this study. Ranges in brackets indicate alternative lifetimes making different assumptions as discussed in the Supplemental Methods (S1 Text).
(DOCX)

S1 Movie Animation of PM_2.5_ Concentrations. Movie showing the evolution of PM_2.5_ concentrations, as inferred by this study, across Eastern China during the four month study interval. Concentrations are shown using color gradients and contour lines; the colors (green, yellow, etc.) represent US EPA qualitative health impacts based on 24-hour exposure. Green is “Good”, yellow is “Moderate”, orange is “Unhealthy for Sensitive Groups”, red is “Unhealthy”, purple is “Very Unhealthy”, and dark red is “Hazardous”. Concentrations higher than the upper limit of the maximum EPA classification are shown in gray.
(MP4)

S1 Fig Average wind and rain patterns in Eastern China. (Left) Average wind speed pattern across China and surrounding areas during the period of this study according to Global Forecast System data at 80 m altitude. Arrow lengths are proportional to wind speed and the legend indicates the size of arrow consistent with a 10 km/hr average wind speed. (Right) Percentage of hours for each grid cell where rain was experienced during the study period based on observations from the Tropical Rainfall Measurement Mission satellite data. Maps were prepared with MATLAB with political boundaries from the Database of Global Administrative Areas (GADM version 2).
(TIF)

S2 Fig Pollution concentration correlation vs. distance. Summary of empirically estimated correlation vs. distance functions for the five pollutants studied. Same values as S2 Table.
(EPS)

S3 Fig Empirical fit of PM_2.5_ correlation data. Heat map of correlation vs. distance generated by all possible pairwise comparisons of PM_2.5_ data from stations in this study. Brighter colors indicate more frequently occurring combinations of R-value and distance, and are normalized for the abundance of comparisons at each distance. The red curve indicates the correlation vs. distance model fit to this data and described in S2 Table.
(EPS)

S4 Fig Impact of assumed plume lifetime on PM_2.5_ source distribution. Maps comparing calculated PM_2.5_ source fluxes for different assumed values of the effective plume lifetime. The upper right panel corresponds to the data reported in the main paper, and has a lifetime chosen such that 5% of the field area is allowed to be negative (same as Fig 4 and S8 Fig). The upper left panel, corresponds to choosing a decay lifetime such that 2% of the flux field is negative, the lower left panel has 15% negative area, and the lower right panel has no plume decay in the absence of rain (infinite lifetime). Rain events are explicitly excluded from consideration, and the reported flux aims to capture the man-made sources of pollution. In general, assuming a shorter plume lifetime implies higher and more diffuse fluxes in order to maintain the observed concentration patterns. By contrast, if the pollutant plumes are assumed to have a very long lifetime then the apparent man-made flux will implausibly turn negative over substantial regions. Though quantitatively important, the choice of effective lifetime has little qualitative impact on the distribution of large sources. The regions of locally higher flux remain similar under all scenarios.
(TIF)

S5 Fig Comparison of PM_2.5_ patterns in early and late half of study. Maps comparing PM_2.5_ concentrations (top) and fluxes (bottom) during the first half of the sampling period (left column) and the second half of the sampling period (right). PM_2.5_ concentrations are presented using the same health impact associated color scheme as Fig 3.
(TIF)

S6 Fig Comparison of PM_2.5_ patterns from half-density reconstructions. The collection of available stations was randomly assigned to two groups, and the analyses for PM_2.5_ concentrations (top) and fluxes (bottom) were repeated in full for both groups. These independent subsamples result in similar concentration and flux patterns, implying the reconstructions are likely to be stable with respect to data selection.
(TIF)

S7 Fig Average PM_2.5_ concentration across China. Larger version of panel from Fig 3 presenting PM_2.5_ concentrations across China. Concentrations are shown using color gradients and contour lines; the colors (green, yellow, etc.) represent US EPA qualitative health impacts. Based on 24-hour exposure green is “Good”, yellow is “Moderate”, orange is “Unhealthy for Sensitive Groups” and red is “Unhealthy”.
(EPS)

S8 Fig PM_2.5_ sources across China. Larger version of panel from Fig 4 presenting PM_2.5_ source fluxes across China.
(EPS)

S9 Fig Average PM_10_ concentration across China. Larger version of panel from Fig 3 presenting PM_10_ concentrations across China. Concentrations are shown using color gradients and contour lines; the colors (green, yellow, etc.) represent US EPA qualitative health impacts. Based on 24-hour exposure green is “Good”, yellow is “Moderate”, orange is “Unhealthy for Sensitive Groups” and red is “Unhealthy”.
(EPS)

S10 Fig PM_10_ sources across China. Larger version of panel from Fig 4 presenting PM_10_ source fluxes across China.
(EPS)

S11 Fig Average SO_2_ concentration across China. Map of SO_2_ concentrations across China. Concentrations are shown using color gradients and contour lines; the colors (green, yellow, etc.) represent US EPA qualitative health impacts. Based on 24-hour exposure green is “Good”, yellow is “Moderate”, orange is “Unhealthy for Sensitive Groups” and red is “Unhealthy”. The limited coverage of this map is a consequence of the short correlation length for SO_2_ (S2 Table; S2 Fig), which limits the distance over which one can usefully interpolate SO_2_. In part, the short correlation length is a consequence of the short lifetime of SO_2_ in the atmosphere, which makes estimates far from measurement sites highly uncertain.
(EPS)

S12 Fig SO_2_ sources across China. Larger version of panel from Fig 4 presenting SO_2_ source fluxes across China.
(EPS)

S13 Fig Average NO_2_ concentration across China. Map of NO_2_ concentrations across China. Concentrations are shown using color gradients and contour lines; the colors (green, yellow, etc.) represent US EPA qualitative health impacts. Based on 24-hour exposure green is “Good”, yellow is “Moderate”, orange is “Unhealthy for Sensitive Groups” and red is “Unhealthy”.
(EPS)

S14 Fig NO_2_ sources across China. Larger version of panel from Fig 4 presenting NO_2_ source fluxes across China.
(EPS)

S15 Fig Average O_3_ concentration across China. Larger version of panel from Fig 3 presenting O_3_ concentrations across China. Concentrations are shown using color gradients and contour lines; the colors (green, yellow, etc.) represent US EPA qualitative health impacts. Based on 24-hour exposure green is “Good”, yellow is “Moderate”, orange is “Unhealthy for Sensitive Groups” and red is “Unhealthy”.
(EPS)

S16 Fig O_3_ sources across China. Map of O_3_ source fluxes across China.
(EPS)
