# Supplementary material for: Air Pollution in China: Mapping of Concentrations and Sources
Source: PLoS One. 2015 Aug 20;10(8):e0135749. doi: 10.1371/journal.pone.0135749 (PMC4546277; doi:10.1371/journal.pone.0135749)
Supplement: S1 Text — Additional discussion of methods, data handling, and validation. (DOCX) [file pone.0135749.s001.docx]

Air Pollution in China: Mapping of Concentrations and Sources

Robert A. Rohde^1,*^, Richard A. Muller^1,2^

Supplementary Methods:

**Air Quality Standards**

For the purposes of qualitatively describing pollution impacts to health, we used the framework and standards adopted by the United States Environmental Protection Agency (US EPA) (*1*). The threshold values for acceptable air quality adopted by the US EPA are similar to those recommended by the World Health Organization (WHO) (*2*), though they differ in some of the details. For example, the WMO recommends PM_2.5_ exposure limits be 30% lower than the allowable value in the US. China has also adopted air quality standards (GB 3095-2012) (*3*). However, the Chinese standards are in general more tolerant of urban air pollution. In the case of PM_2.5_, the acceptable level under Chinese regulations for urban areas is more than double the US standard and three times the WMO recommended value. For the figures in this paper, we used the US air quality labels and colors.

**Air Quality Data**

Due to download restrictions on China’s official reporting platform (http://113.108.142.147:20035/emcpublish/), two different third-party portals were used. The first, PM25.in, is a direct mirror of the real-time data from the 945 Chinese national network sites. The second, AQICN.org, is the largest global aggregator of real-time air quality data, including observations from more than 4500 monitoring stations via approximately 100 data providers. AQICN, which was originally founded with a focus on Asia, reports 1599 real-time monitoring sites in China and Taiwan attributed variously to the Chinese national network, 21 Chinese provincial environmental agencies, the Taiwan Environmental Protection Agency, as well as five stations operated by the US government at embassy or consulate locations. In addition, AQICN provides an additional 242 sites in territories within 500 km of China, which can be used to help constrain pollutant concentrations in border regions. The use of two data providers allowed for continuity if either single data source became unavailable (as did occur several times during our data collection interval). Further, it also allowed the sources to be compared for consistency. Such consistency checks revealed a problem with the AQICN data for CO, and as a result CO was omitted from the analysis in this study.

Though nominally each site may report every hour, on average new data was found on 85% of hours. To simplify our analysis, sites active for fewer than 30% of possible hours were excluded. In addition, a review of station correlations strongly suggested that some station locations were misreported, e.g. data was poorly correlated with other measurements of the same area but highly correlated with another region far away (often 100s of km from the nominal location). In general, all station location data was derived from the location provided by AQICN. It is unclear how precise these locations should be expected to be. Though precise coordinates may be available for some stations, the locations for some stations were assigned via geocoding (i.e. looking up named features on a map). In many cases, station names do identify a specific facility at which a station is located (e.g. “Caotang Temple, Chengdu”, “Three Rivers Department of Transportation, Langfan”); however, in other cases a station only lists the city it is placed in. It is likely therefore that some station locations are rather imprecise or erroneous. After reviewing station correlations, 33 stations were excluded from the analysis due to probable large location reporting errors.

**Data Quality Control**

Automated quality control checks were performed on each air pollutant to remove repeated values, overly frequent values, and implausible zeroes, common signs of missing measurements being replaced with a placeholder. Collectively, these quality control checks excluded 7.7% of the reported values from China and Taiwan. The most common exclusion criterion (58% of exclusions) was for pollutants reported to have the same non-zero concentration for several hours in a row. Due to noise and natural variability, instruments will rarely give the same value two hours in a row. If the reading is the same for many hours, it is more likely that the instrument has stopped operating and the reporting system is simply repeating the last valid measurement received. In some cases, repeated values continued every hour for as long as 3 weeks.

In addition, a regional consistency check was performed for each pollutant where the value at each location was compared to the average value at nearby stations at the same time. The least consistent 0.5% of observations were excluded. In many cases, the observations removed are more than double what one would expect based on the average of their neighbors. Data removed as a result of this test is not necessarily inaccurate, but may reflect a pollution source in the immediate vicinity of the monitoring site that is not representative of conditions in the rest of the city or region. As we wish to focus on large-scale patterns, it is desirable to remove such local outliers. Additional steps to compensate for local noise and outliers were built into the interpolation scheme described below. In addition to these quality control steps, data gaps in a station record lasting two hours or less were infilled by linear interpolation to help reduce the noise in the reconstruction due to missing stations.

**Weather Data**

In order to analyze sources and flow patterns, we needed data on wind and rain for the hours covered. We obtained the wind information from the Global Forecast System (GFS) (*4*). Wind fields are provided at 0.5 degree resolution and 6 hour time steps; we used linear interpolation on the data to obtain the required 1-hour intervals and 6 km spacing. For the present work, emphasis was given to short-term changes in near-surface conditions and the wind field at 80 m above ground was selected. In general, one expects air pollutants to be approximately well-mixed within the planetary boundary layer (PBL), which is the layer of air near the surface that is mixed by convection and turbulence (*5*). The height of the planetary boundary layer varies with location, season, weather, and hour of day with typical heights from 200 m to 2000 m (*6*). GFS also provides wind layers at 2 m and 100 m above ground. Tests using these alternative wind heights in flux reconstructions were also conducted, and the impact was found to be negligible. For example with PM_2.5_, the typical root-mean-square difference in the estimated flux associated with using an alternative wind layer was less than 1% of the mean flux.

Average wind patterns during the study are shown in Figure S1. In the Northern most parts of China there is on average a prevailing West to East / North to South flow pattern, which will carry pollution out to sea. In Central and Southern China the prevailing wind pattern is weaker, and often averages East to West, which could cause coastal pollution to move inland. In the region just South of Beijing, the wind flow patterns averaged South to North, implying the air pollution from the industrial regions South of Beijing were likely to impact the capital during the period of this study.

As air pollution is influenced by precipitation, rain observations were also used. Most of the analysis was done with TRMM satellite estimates of rainfall (*7,8*), and have 0.25 degree resolution and three hour time steps (data set 3B42RT). For locations and times when TRMM data was unavailable (roughly 0.06% of all needed data), GFS rain estimates were used instead. For the present study, a simple rain / no rain classification was constructed at each location and time. Precipitation rates were linearly interpolated and fluxes exceeding 0.2 mm/hr were classified as rain cells. A nonzero threshold was chosen to avoid over reporting trace amounts of rain due to interpolation. The percentage of hours classified as “rain” is shown in Figure S1.

**Population and Location Data**

In order to access spatial variations in health impacts, the GADM database of Global Administrative Areas (version 2; http://gadm.org/) was used to define province and prefecture level divisions in China. The average pollutant concentration and flux was calculated by averaging over each defined region. Due to a few errors and out-of-date definitions (dating from roughly 2012), a few of these regions differ from the present configuration of political divisions in China. Notes on differences are included with the tabular data (Table S1). Population data at the prefecture level was taken from the 2010 census of China. Using the GADM classifications, China has 345 prefectures. All statements related to the size of populations affected by pollution were calculated at the prefecture level and do not include Taiwan.

**Spatial Interpolation (Kriging)**

This section provides a summary of the interpolation process used to derive pollution maps and time series. Mathematical formalism is provided in the following section.

After removing outliers, each pollutant was subjected to Kriging interpolation (also known as Gaussian process regression) to produce a continuous concentration field across Western China during each hour in the study period. For the analysis described in this paper, this continuous field was sampled with an approximately 6 km resolution. Though computed at a 6 km resolution, in practice, the characteristic size of resolvable features is often larger (e.g. 30 km) and varies with station density and noise.

The Kriging process was accomplished in two steps. First, an average value field was constructed by applying Kriging (*9-12*) to estimated mean concentrations of the pollutant at each measurement site using latitude, longitude, and their second order products as additional predictive variables. Secondly, at each location and each hour, an anomaly field was created by applying simple Kriging to the differences between the current observation and the mean value for that station. The concentration at each time and location was then estimated as the sum of the time-invariant average value field and the time-varying anomaly field. The use of this two-step process helped reduce reconstruction errors associated with missing values.

The covariance matrices required by Kriging were estimated using empirically determined correlation versus distance functions modeled as two part exponential sums (see Table S2, Figure S2, and Figure S3). The empirical correlation functions were found by computing the correlation between all possible pairs of stations used in this study, plotting the resulting correlations vs. distance and then fitting a linear combination of exponential functions. An example of this is shown in Figure S3 for PM_2.5_. The choice of functional form to use is somewhat arbitrary and was based on the desire to obtain a good functional fit with relatively few parameters, though the set of known functions that generate valid correlation matrices is also a restriction (*13,14*).

For each pollutant, the correlation vs. distance function generally contains a short-range term (40 – 50 km) that likely reflects heterogeneity in the source distribution, and a long-range term (250 – 600 km) that probably reflects weather patterns. The relative importance of each is influenced by the residence time in the atmosphere of the pollutant and the discreteness of its sources. SO_2_ has by far the sharpest correlation function, driven by the combination of a short residence time and highly discrete sources (mostly coal burning power plants and industrial facilities). By contrast, PM_2.5_ and PM_10_ have broad correlation functions consistent with the relatively long residence time of particulate matter. The shape of the correlation function directly determines the distance over which Kriging interpolations and extrapolations are plausibly useful. As a result of its sharper correlation function, the same set of stations will tell us less about SO_2_ distributions than other pollutants.

An additional feature of the correlation function, highlighted in Table S2, is the correlation at zero distance, *R*(0). With perfect data one would expect *R*(0) ≈ 1, but this diverges from unity as a result of “noise” in the data, including instrumental noise, errors in reported station location, and ultra-local pollution sources that don’t significantly impact more than one station. The fraction of variance in the typical station record attributable to such “noise” can be estimated as 1 – *R*(0). The analysis framework was intentionally designed to allow for *R*(0) < 1 as a method of compensating for noisy and erroneous data. With *R*(0) < 1, the interpolated field will not exactly match the observations at a station location, but instead the field will be somewhat smoother in a way that attempts to compensate for the typical level of noise in the observations. Along with the quality control steps described above, this approach plays an important role in controlling for potential problems in the data set.

**Mathematical Formalism**

The following section provides the mathematical formalism for the Kriging procedure used.

The pollutant field, $P(\vec{x},t)$ is decomposed into a stationary part, $S\left(\vec{x}\right)()$, and a time varying anomaly part, $A\left( \vec{x},t \right)$:

The stationary part is modeled using Kriging with latitude, *λ*, and longitude, *φ*, as global predictive variables.

Where $\overline{p_{n}}$ denotes the estimated mean pollutant concentration at site *n*, the global auxiliary function $G\left(\vec{x}\right)()$ is determined by parameters *α*, *β*, *γ*, *ε*, *η*, and *κ* chosen to minimize the average Kriging error over the field and Kriging coefficients, $K_{n}\left(\vec{x}\right)()$, are defined by:

Here is given by the correlation vs. distance model described in Table S2, e.g. $R\left( x_{1},x_{2} \right)=R\left( \left\| x_{1}-x_{2} \right\| \right)$. Note that the matrix diagonal elements are defined to be unity, even though the *R*(0) values in the correlation model are not. This difference incorporates the notion of noise (e.g. added variance) at each location. The typical Kriging construction uses covariance rather than correlation. Correlation and covariance constructions are equivalent if the variance is the same at all locations, and remain approximately equivalent as long as the variance varies slowly with position. The correlation approach is chosen for convenience. A similar approach was used in (*15*).

The time-varying anomaly portion of the pollutant field is defined as:

Where $p_{n}\left( t \right)$ is the pollutant concentration at time *t* for site *n*, and the Kriging coefficient $K_{n}^{*}\left( \vec{x_{n}},t \right)$ is calculated the same as $p_{n}\left( t \right)$ $K_{n}\left( \vec{x} \right)$except that only sites that are active at time *t* are included in the matrix equation for . By recomputing the Kriging coefficients at each time step a more accurate field is achieved that more effectively adjusts for missing data.

**Mean pollutant concentration and regional outliers**

If data exists for every hour, then $\bar{p_{n}}$is simply defined as the time average of $p_{n}\left( t \right)$. However, for most sites multiple hours are missing. In order to estimate $\overline{p_{n}}$ in an unbiased way, it is therefore necessary to estimate the pollutant concentration during hours when no data are available. This is done by assuming that $p_{n}\left( t_{\mathrm{missing}} \right)\approx P\left( \vec{x_{n}},t_{\mathrm{missing}} \right)$. is computed as described above by initially estimating $\overline{p_{n}}$ from the available observations and iteratively adjusting the estimated $\overline{p_{n}}$ using to replace missing values. This iterative process quickly converges and helps avoid the risk of biasing $\overline{p_{n}}$ at sites with large blocks of missing data.

In a similar way, we estimate regional consistency by measuring the misfit $\left( p_{n}\left( t \right)-P\left( \vec{x_{n}},t \right) \right)^{2}$. These misfit values are collected for all times and sites and then sorted. The largest 0.5% of misfits are presumed as likely outliers. Initially such outliers are replaced with $P\left( \vec{x_{n}},t \right)\pm M$ where *M* denotes the largest misfit not excluded and the sign is taken in the same direction as the outlier. This initial replacement helps ensure numerical stability, and the process is iterated until it converges on a stable set of outliers. The final set of outliers is then discarded before computing a final solution for $P\left( \vec{x},t \right)$. The outliers removed in this way are not necessarily erroneous reports, but in many cases may indicate an ultra-local pollution source in the immediate vicinity of the monitoring station that causes large excursions at that site but does not have a large impact at other locations. It is not uncommon for pollutant concentrations flagged as outliers to more than double the regionally expected concentration. Removing these ultra-local effects allows for better regional-scale estimates, though it may mean that the impact of pollution in the immediate vicinity of a monitoring station is sometimes understated.

It must also be noted that this regional outlier detection scheme will only work effectively when stations are highly clustered (several stations within ~40 km). If there is only one station in an area, then it will always be true that $P\left( \vec{x_{n}},t \right)\approx p_{i}\left( t \right)$, and no regional outlier detection is possible. Here, the high degree of redundancy in the Chinese station network (e.g. multiple stations per urban area), is likely a considerable benefit.

**Source Determination**

There are several chemical transport models for predicting the formation and evolution of air pollution as an extra layer on top of a weather model, such as GEOS-Chem (*16*) and CMAQ (*17*). Though they can be effective, such codes generally require a considerable investment of computational power, operator time, and detailed information about weather conditions in and around the study area. In most applications, they also tend to have crude resolution (e.g. 0.5 degrees). Rather than adopt an existing weather model, we chose to apply a simpler framework that relied only on short-term changes in the air pollution field and a limited input of weather data. This is possible because of the high level of spatial and temporal detail provided by the Chinese air quality network.

To estimate surface fluxes we look at short-term transport process and hour-by-hour changes in pollutant concentration. We begin with the set of interpolated pollution maps,$P(\vec{x},t)$ , constructed as described above for every hour. Given a wind field, $\vec{v}(\vec{x},t)$, one can predict that the pollution field will evolve according to the wind, such that:

By comparing the map one would expect due to wind flow to the map of pollution that is directly observed at $t+\Delta t$, one can estimate the pollution fluxes that must have occurred during the interval $\Delta t$, as:

Further improvements can be made by considering both forward and reverse evolutions, and by including consideration of the pollution losses due to other processes, such as dry settling and/or precipitation. The incorporation of forward and reverse evolutions, as well as an effective lifetime, *τ*, leads to our estimate of the pollution flux:

In the limit of *τ* goes to infinity, this reduces to the secant method for estimating the derivative of $P\left( \vec{x},t \right)$ with respect to time. The use of both forward and reverse flow terms is desirable as it allows for the partial cancelation of errors resulting from inaccuracies in the pollution reconstruction and/or wind field. The field motion terms, represented as $\vec{x}\pm v\left( x,t \right)*\Delta t$, were actually computed by numerical integration to give a more accurate evolution such that

The effective lifetime, *τ*, is intended to approximately summarize all of the non-precipitation processes that cause near-surface pollutant concentrations to change over time, including chemical reactivity, dry deposition, and vertical mixing. This formalism assumes that in the absence of sources, the pollution concentration would decay exponentially, e.g. $P\left( t+\Delta t \right)\approx P(t)e^{\frac{-\Delta t}{\tau}}$. Expressing all of this via a single effective lifetime is, of course, a considerable approximation as the actual time evolution of pollutant concentrations is likely to depend on many factors, such as relative humidity, temperature, sunlight, wind speed, and presence of other pollutants, etc. For many of these factors, we have little or no direct information. As discussed below the parameter, *τ*$\tau$, is estimated empirically. Its value has a quantitative impact on the reconstruction, but generally does not qualitatively impact the results.

We note that our formalism for the time evolution of the concentration field contains no diffusion terms, e.g. terms proportional to $\nabla^{2}P(\vec{x},t)$. It is assumed that such terms are effectively negligible on the hourly time scale being considered, and that the only significant mixing is due to wind. Likewise, vertical wind components and changes in pollutant concentration with altitude are not considered. Such assumptions are plausible because the near-surface pollution tends to be well-mixed due to the effects of the boundary layer, and because vertical wind velocities are typically two orders of magnitude lower than horizontal velocities (except during rare episodes of strong convection, such as thunderstorms). To the extent that the neglected processes are important, they will contribute additional noise to the flux estimates.

Consistent with the approach described above, fluxes are expressed as the rate of change in the near-surface pollutant concentration over time (i.e. units of concentration / time). This could be converted to a surface flux (e.g. units of kg / km^2^ / hour) by considering the height of the air column being polluted. This conversion might typically be accomplished by multiplying by the height of the planetary boundary layer, which varies by location, season, and time of day (*6*). For the purposes of the present work we have not tried to make the conversion.

**Flux Averaging**

In principle, all that is needed to estimate the average pollutant flux is to take a time-average of $F\left( \vec{x},t \right)$. However, in practice it is desirable to take some steps to remove outliers and related effects. First, in order to emphasize human-caused pollution fluxes, flux values were ignored from locations and times flagged as containing rain (or rain at the same location on adjacent hours).

To further filter the flux data, we apply a simple outlier detection model. At each location, the flux field is fit to a constant term plus a periodic component that is allowed to vary with the time of day. The latter consideration is important as pollutant fluxes at most locations show diurnal variations. For particulate matter, the diurnal range is small and most likely a consequence of day/night fluctuations in the height of the planetary boundary layer. For NO_2_ and O_3_, the daily fluctuations are much larger and presumably reflect the fact that these gases are photosensitive. The differences from the fit are sorted and the upper and lower 5% are each discarded. The fit is then repeated and the resulting constant term reported as the average flux.

In many cases, the mean with and without exclusion of the extremes is little changed; however, at some locations there are many asymmetric outliers. Extremely negative flux events observed at some locations are probably weather events (e.g. rain) that were not accounted for by the rain classifier. In addition, there are some rare and extremely large particulate matter fluxes in the northwest of China that are associated with desert dust storms. The extra step of removing outlying values before averaging should make flux averages at each location more reflective of the typical man-made component.

**Estimating effective lifetime**

The effective lifetime, *τ*, is used as a crude approximation for decreases in pollutant concentration driven by factors other than precipitation. This is not a chemical lifetime (though chemical reactions may influence it), but is more properly understood as a plume lifetime measuring how quickly a pollutant plume dissipates. For some pollutants, e.g. SO_2_, the effective plume lifetime is probably primarily a function of chemical transformations, but other pollutants, e.g. particulate matter, are likely to be more influenced by settling and vertical mixing.

Many factors can influence plume lifetime, including temperature, relative humidity, wind speed, altitude, particle size and composition, abundance of sunlight, and the presence of other pollutants. Because of these factors, it is challenging to estimate plume lifetimes from first principles. In modern times, complex computer models are often used to study the atmospheric transport of pollutants (*16,17*). However, the use of such models requires either a great deal of data or the willingness to make many assumptions. The flux approach described above is much simpler than typical computer models and makes it easy to provide basic estimates, though it is likely that future studies will improve upon the simple process described here. However, in order to complete even our basic flux estimate, it is still necessary to make some assumption about the background rate at which pollutants dissipate.

To place some constraint on the pollutant plume lifetime, we make an assumption that the lowest man-made fluxes will be approximately zero. In other words, we assume that human activity only adds pollution, and doesn’t directly remove it. This has the effect of incorporating all of the non-rain removal processes into the estimate of *τ*. Given this assumption, we would expect that the resulting flux field will be positive or near-zero nearly everywhere. Of course, some negative values may occur simply due to noisy data, but they would not be expected to be common. By contrast, if we had assumed there is no pollutant dissipation at all (i.e. $\tau=\infty$) then roughly 1/3 of the PM_2.5_ flux field would be negative. Instead, we fix the lifetime parameter such that exactly 5% of the flux field is negative.

Across the different pollutants, a 5% criterion results in effective plume lifetimes that run from 1.9 days to 5.5 days (Table S2). Also provided in Table S2 are estimates of the parameter value corresponding to allowing 2% or 15% of the area having negative values. Though we can reasonably expect that the flux will be positive nearly everywhere, the choice of threshold to use is somewhat arbitrary. However, as discussed below, the choice of threshold does not have a large qualitative effect on the distribution of sources. This is expected since man-made sources tend to be large in magnitude but spatially compact, while dissipation is slower and occurs over a much larger area. For PM_2.5_, the flux at the largest sources exceeds the losses associated with the typical dissipation rate by more than an order of magnitude. As a result, plausible errors in the dissipation rate can have only a modest impact on the anthropogenic source distribution.

This approach of estimating an effective plume lifetime is reasonable provided that the natural fluxes are often negligible, the lifetime is approximately uniform across the field, and at least 10% China’s area is reasonably free from man-made emissions. If there are significant natural sources or significant spatial heterogeneity in lifetime, this estimate of an effective lifetime may be too short. In general, using a lifetime that is too short will result in overestimating man-made fluxes. Though errors in estimating the effective lifetime are quantitatively important, they do not tend to change the qualitative distribution of sources. In particular, such an error tends to increase or decrease the apparent flux nearly everywhere at the same time. To demonstrate the effect, Figure S4 shows the resulting PM_2.5_ flux pattern given a range of assumed lifetimes.

**Stability of inferred concentrations and fluxes**

Several tests were conducted to determine whether the reported concentration and flux patterns were consistent over the duration of the study or whether they changed significantly. Figure S5 shows a comparison of the PM_2.5_ concentration and flux from April 5 to June 4 (first half of study interval) and then from June 5 to August 5 (second half of study). There are some differences, especially in the western half of the study region, where pollutant concentrations were 10 – 20 μg/m^3^ higher during the first part of the study then the latter portion. However, the panels are more similar than they are different, with extensive pollutant patterns and similar flux distributions in each half. This leads us to believe that the patterns are likely to be relatively stable over the long-term, though it will be interesting to study seasonal variations in the future. As discussed in the main paper, the limited evidence available suggests that average concentrations over the four months of the current study are not greatly different from concentrations that occur annually. Ultimately it will be important to explore time variations in air pollution patterns, e.g. seasonality and long-term trends, but such studies will require a longer record than used in the present study.

**Split Sample Reproducibility Test**

In order to test the impact of variations in station network composition, each station was randomly assigned to one of two groups, and each group was separately reconstructed for PM_2.5_ flux and concentration. All major steps in the analysis were repeated independently for each analysis block, including the determination of the correlation vs. distance function, and the determination of the effective lifetime. Figure S6 shows the results. The reconstructions from these independent data sets are similar to each other and to the total reconstruction, indicating that the present analysis is not very sensitive to the specific stations included. In large part, the similarity between the half-density reconstructions is likely a consequence of China’s habit of having multiple stations clustered in most urban areas, so that there is a large measure of redundancy.

To the extent that there are differences in the split sample reconstructions, they can provide us some insight into the uncertainty of the reconstruction. The maximum difference in concentration is 22.8 μg/m^3^ with a root mean square difference of 4.0 μg/m^3^. This suggests that the typical 1-sigma uncertainty in the full reconstruction is probably around $\frac{4.0}{\sqrt{2}}=2.8$μg/m^3^. Direct estimation using formal Kriging errors gives a similar value of 2.6 μg/m^3^ for the typical PM_2.5_ average concentration uncertainty. However, as illustrated in Figure S6, one should be aware that the actual error may be larger in some regions rather than others. A similar comparison of the flux panels suggests that the typical flux uncertainty may be around 0.18 μg/m^3^/hr, though a few spots suggest locally larger uncertainties.

**Comment about Negative Flux Values and Zhangjiakou**

Due to noise and the limitations of our modeling approach we expect there to be some regions where the estimated anthropogenic flux of pollutants is negative. In many cases, negative apparent values are probably just noise at sites where the true flux is near zero. In a smaller percentage of cases, perhaps impacting 1-2% of China’s area, larger negative values may reflect the limitations of our data and/or our modeling assumptions. For example, systematically bad data may give rise to biased results. The quality control and interpolation scheme should compensate for many errors but may not completely eliminate all issues. In addition, the flux modeling may be limited due to spatial variations in plume lifetime or large impacts from vertical mixing or other effects not explicitly considered in the analysis described here.

The urban core of Zhangjiakou is the most prominent location on the PM_2.5_ flux map with a very large negative flux. Due to the magnitude of this negative anomaly, we investigated the underlying data and analysis process in this region to look for problems. Our conclusion is that the large negative flux is consistent with the data reported from Zhangjiakou; however, the pollution concentrations reported from this location are suspicious. There are four monitoring stations in the urban core of Zhangjiakou with sufficient data to be included in this study. When compared to stations in outlying regions at distances of 20-80 km, each station in the city averaged only about half as much PM_2.5_ pollution as the outlying areas. Further, several additional monitoring sites have recently been added near Zhangjiakou. These additional stations are too recent to be included in the present paper, but they also appear to show the higher levels of pollution suggested by outlying areas. We are left to conclude that either the city of Zhangjiakou consistently and surprisingly experiences locally cleaner air than nearby areas, or that the stations in the city are systematically reporting pollution levels that are erroneously low. We believe the latter scenario is more likely, though on the ground inspection and validation of the Zhangjiakou monitoring sites would be necessary to know for sure.

**Estimation of Deaths Associated with Air Pollution**

There are substantial uncertainties regarding the health impact of air pollution. Large studies conducted primarily in developed countries have estimated the increase in all-cause mortality due to air pollution, with a variety of results. Pope et al. (*18*) gave an estimate of +4% mortality per 10 μg/m^3^ of PM_2.5_ (95% confidence interval: 1-8% per 10 μg/m^3^). More recently, Hoek et al. (*19*) estimated +6 % per 10 μg/m^3^ (4-9% confidence range), and Beelen et al. (*20*) estimated +14% per 10 μg/m^3^ (4-26% confidence range). For the current work, we adopt the multi-component model for PM_2.5_ impacts used by the World Health Organization (*21*, *22*). This model considers five distinct disease classes and allows for non-linearity in response as a function of pollutant concentration (*21*). The five diseases considered are stroke, ischemic heart disease, lung cancer, chronic obstructive pulmonary disease, and lower respiratory infection (*21*).

To calculate the change in mortality due to air pollution, the relative risk of death $RR$ is estimated for each of the five diseases as a function of PM_2.5_ concentration using numerical tables provided from Burnett et al. (*21*). For each disease, the population attributed fraction is then calculated by summing over prefectures:

$$PAF=\frac{\sum P_{i}(RR_{i}-1)}{\sum P_{i}RR_{i}}$$

Where *P_i_* is the prefecture population and *RR_i_* is the relative risk of death in the prefecture given the observed average pollution concentration. The counterfactual relative risk is assumed to be unity, consistent with the way the integrated response model was designed. The total associated mortality is then estimated by scaling the PAF by the estimated total number of deaths from each modeled disease in China as reported in the Global Burden of Disease study (*22*). For the purposes of the current estimate we have not attempted to account for age or sex dependencies in relative risk, and we have assumed that the separate causes of death can be regarded as independent. Numerical details of the calculation appear in Table S1.

The aggregate total for air pollution related deaths was calculated to be 1.6 million deaths/year [95% confidence range 0.7 − 2.2 million deaths per year]. This death total implies that PM_2.5_ air pollution contributes to approximately 17% of all deaths in China. Approximately half of the attributed deaths are attributed to an increased risk of stroke. A further 25% of deaths are associated with ischemic heart disease, and the balance of excess mortality is distributed amongst the other three diseases considered.

**US Mission Stations Disclaimer**

The dataset analyzed in this paper included data from five air quality stations operated by the US Department of State at US embassy and mission facilities in China. The US Department of State requires that the following disclaimer by included in any publication that uses these data:

State Air observational data are not fully verified or validated; these data are subject to change, error, and correction. The data and information are in no way official.

References:

1. US EPA. Air Quality Index: A Guide to Air Quality and Your Health. U.S. EPA Report EPA-456/F-14-002. Available: <http://www.epa.gov/airnow/aqi_brochure_02_14.pdf>
2. World Health Organization. (2006) *WHO Air quality guidelines for particulate matter, ozone, nitrogen dioxide and sulfur dioxide: Global update 2005.* (World Health Organization Report WHO/SDE/PHE/OEH/06.02, 2006; whqlibdoc.who.int/hq/2006/WHO_SDE_PHE_OEH_06.02_eng.pdf)
3. Government of China. (2012) Ambient Air Quality Standards (in Chinese). GB 3095-2012. Available: http://kjs.mep.gov.cn/hjbhbz/bzwb/dqhjbh/dqhjzlbz/201203/W020120410330232398521.pdf
4. NOAA/NCEP. *Global Forecast System (GFS) Atmospheric Model.* (2012; www.ncdc.noaa.gov/data-access/model-data/model-datasets/global-forcast-system-gfs)
5. Lin J-T, McElroy MB. (2010) Impacts of boundary layer mixing on pollutant vertical profiles in the lower troposphere: Implications to satellite remote sensing. *Atmos. Env.* **44**, 1726-1739.
6. von Engeln A, Teixeira J. (2013) A Planetary Boundary Layer Height Climatology Derived from ECMWF Reanalysis Data. *J. Clim.* **26**, 6575–6590.
7. Huffman GJ, Bolvin DT, Nelkin EJ, Wolff DB, Adler RF, et al. (2007) The TRMM Multi-satellite Precipitation Analysis: Quasi-global, multi-year, combined-sensor precipitation estimates at fine scale. *J. Hydrometeor*. **8**(1), 38-55.
8. Huffman GJ, Bolvin DT. (2011) *Real-Time TRMM Multi-Satellite Precipitation Analysis Data Set Documentation*. NASA/GSFC Laboratory for Atmospheres, 43 pp. Available: ftp://meso.gsfc.nasa.gov/pub/trmmdocs/rt/3B4XRT_doc.pdf.
9. Schabenberger O, Gotway CA. (2004) *Statistical Methods for Spatial Data Analysis.* CRC Press. ISBN 020349198X.
10. Schuenemeyer J, Drew L. (2011) *Statistics for Earth and Environmental Scientists.* John Wiley and Sons. ISBN 1118102215.
11. Krige DG. (1951) A statistical approach to some basic mine valuation problems on the Witwatersrand. *J Chem Metall Min Soc S Afr.* December: 119-159.
12. Matheron G. (1963) Principles of geostatistics. *Econ. Geol.* **58**:1246-1266.
13. Gneiting T. (2013) Strictly and non-strictly positive deﬁnite functions on spheres. *Bernoulli* **19**(4): 1327–1349.
14. Huang C, Zhang H, Robeson SM. (2011) On the validity of commonly used covariance and variogram functions on the sphere. *Math. Geosci.* **43**(6): 721–733.
15. Rohde RA, Muller RA, Jacobsen R, Perlmutter S, Rosenfeld A, et al. (2013) Berkeley Earth Temperature Averaging Process. *Geoinfor Geostat: An Overview* **1**(2) doi:10.4172/gigs.1000103.
16. Bey I, et al. (2001) Global modeling of tropospheric chemistry with assimilated meteorology: Model description and evaluation, *J. Geophys. Res.* **106**: 23073-23096.
17. Foley KM, et al. (2010) Incremental testing of the Community Multiscale Air Quality (CMAQ) modeling system version 4.7, *Geosci. Model Dev.*, **3**(1): 205-226.
18. Pope CA, Burnett RT, Thun MJ, Calle EE, Krewski D, et al. (2002) Lung cancer, cardiopulmonary mortality, and long-term exposure to fine particulate air pollution. *JAMA* **287**(9): 1132–1141. doi:10.1001/jama.287.9.1132. PMID 11879110.
19. Hoek G, Krishnan RM, Beelen R, Peters A, Ostro B, Brunekreef B, Kaufman JD (2013) Long-term air pollution exposure and cardiorespiratory mortality: a review. *Env. Health*. **12**:43.
20. Beelen R, Raaschou-Nielsen O, Stafoggia M, Andersen ZJ, Weinmayr G (2013) Effects of long-term exposure to air pollution on natural-cause mortality: an analysis of 22 European cohorts within the multicentre ESCAPE project. *Lancet* **383**(9919): 785-795.
21. Burnett RT, Pope CA III, Ezzati M, Olives C, Lim SS, Mehta S, Shin HH, Singh G, Hubbell B, Brauer M, Anderson HR, Smith KR, Balmes JR, Bruce NG, Kan H, Laden F, Prüss-Ustün A, Turner MC, Gapstur SM, Diver WR, Cohen A. (2014) An integrated risk function for estimating the global burden of disease attributable to ambient fine particulate matter exposure. *Environ Health Perspect* 122:397–403; http://dx.doi.org/10.1289/ehp.1307049
22. Naghavi M, Wang H, Lozano R, Davis A, Liang X, Zhou M, et al. (2015) Global, regional, and national age–sex specific all-cause and cause-specific mortality for 240 causes of death, 1990–2013: a systematic analysis for the Global Burden of Disease Study 2013. The *Lancet* 385 (9963): 117–171.
