# Supplementary material for: Air Pollution in China: Mapping of Concentrations and Sources
Source: PLoS One. 2015 Aug 20;10(8):e0135749. doi: 10.1371/journal.pone.0135749 (PMC4546277; doi:10.1371/journal.pone.0135749)
Supplement: S2 Table — Summary of the empirically determined correlation vs. distance functions for the five pollutants discussed in this study and the effective residence time in the atmosphere of their pollutant plumes in the absence of rain or snow. The correlation functions generally consist of a short range component (influenced largely by source distributions) and a long-range component (influenced by weather patterns). The effective pollutant plume lifetime shows the value empirically estimated for this study. Ranges in brackets indicate alternative lifetimes making different assumptions as discussed in the Supplemental Methods (S1 Text). (DOCX) [file pone.0135749.s003.docx]

| Pollutant | Correlation vs. Distance | *R*(0) | Effective Pollutant Plume Lifetime (days) |
| --- | --- | --- | --- |
| PM_2.5_ | $0.23e^{-d/(48 km)}+0.61e^{-d/(350 km)}+0.03$ | 0.87 | 5.4 [3.8 – 11.7] |
| PM_10_ | $0.19e^{-d/(50 km)}+0.64e^{-d/(290 km)}+0.09$ | 0.92 | 4.1 [2.9 – 9.2] |
| SO_2_ | $0.42e^{-d/(48 km)}+0.15e^{-d/(910 km)}+0.01$ | 0.59 | 1.9 [1.3 – 4.5] |
| NO_2_ | $0.36e^{-d/(48 km)}+0.31e^{-d/(540 km)}+0.09$ | 0.76 | 3.8 [2.4 – 7.7] |
| O_3_ | $0.07e^{-d/(42 km)}+0.54e^{-d/(310 km)}+0.24$ | 0.86 | 5.5 [3.3 – 11.1] |
