# Supplementary figures and images for: Air Pollution in China: Mapping of Concentrations and Sources
Source: PLoS One. 2015 Aug 20;10(8):e0135749. doi: 10.1371/journal.pone.0135749 (PMC4546277; doi:10.1371/journal.pone.0135749)

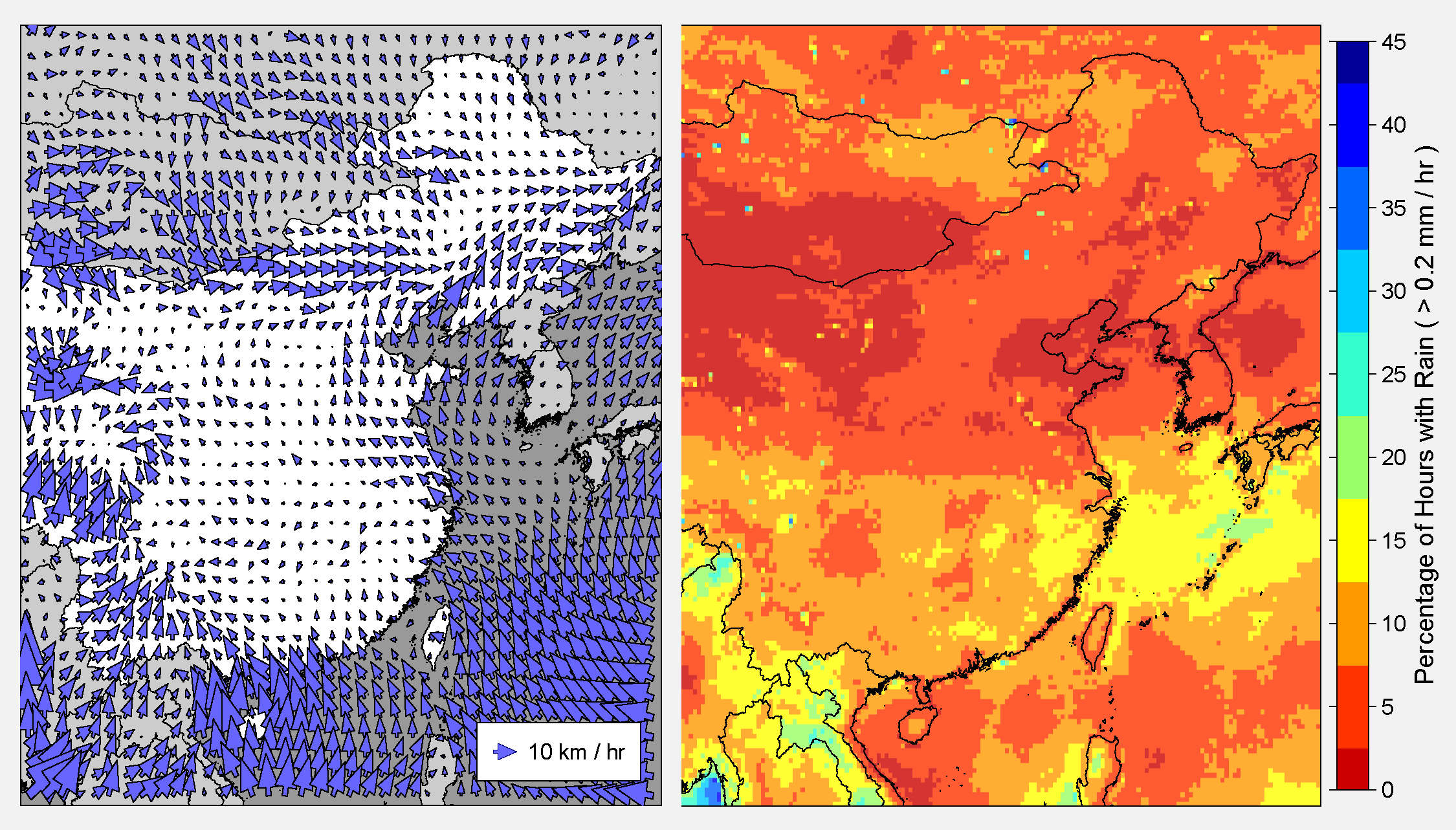

Supplement: S1 Fig — (Left) Average wind speed pattern across China and surrounding areas during the period of this study according to Global Forecast System data at 80 m altitude. Arrow lengths are proportional to wind speed and the legend indicates the size of arrow consistent with a 10 km/hr average wind speed. (Right) Percentage of hours for each grid cell where rain was experienced during the study period based on observations from the Tropical Rainfall Measurement Mission satellite data. Maps were prepared with MATLAB with political boundaries from the Database of Global Administrative Areas (GADM version 2). (TIF) [file pone.0135749.s005.tif]

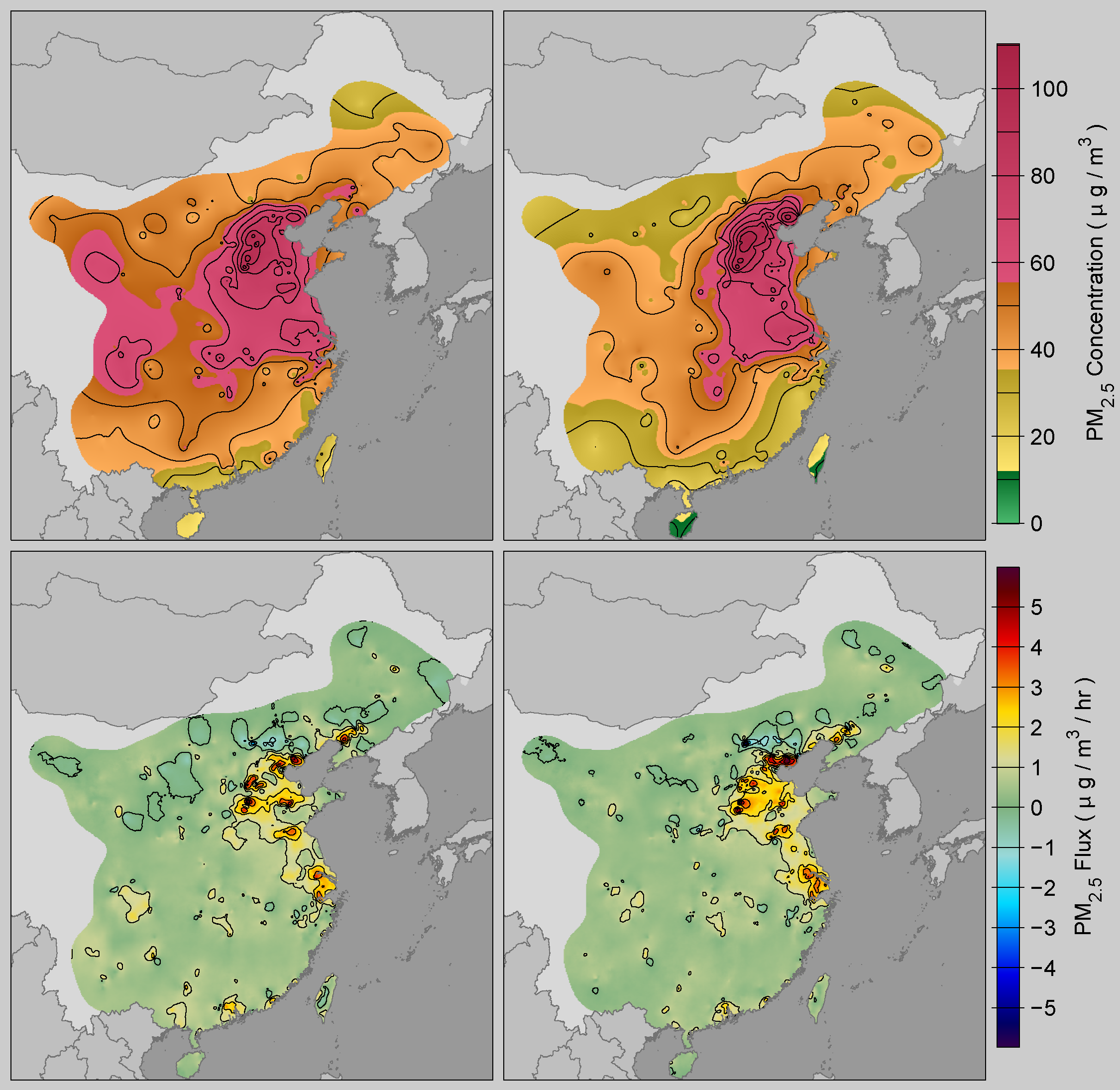

Supplement: S4 Fig — Maps comparing calculated PM2.5 source fluxes for different assumed values of the effective plume lifetime. The upper right panel corresponds to the data reported in the main paper, and has a lifetime chosen such that 5% of the field area is allowed to be negative (same as Fig 4 and S8 Fig). The upper left panel, corresponds to choosing a decay lifetime such that 2% of the flux field is negative, the lower left panel has 15% negative area, and the lower right panel has no plume decay in the absence of rain (infinite lifetime). Rain events are explicitly excluded from consideration, and the reported flux aims to capture the man-made sources of pollution. In general, assuming a shorter plume lifetime implies higher and more diffuse fluxes in order to maintain the observed concentration patterns. By contrast, if the pollutant plumes are assumed to have a very long lifetime then the apparent man-made flux will implausibly turn negative over substantial regions. Though quantitatively important, the choice of effective lifetime has little qualitative impact on the distribution of large sources. The regions of locally higher flux remain similar under all scenarios. (TIF) [file pone.0135749.s008.tif]

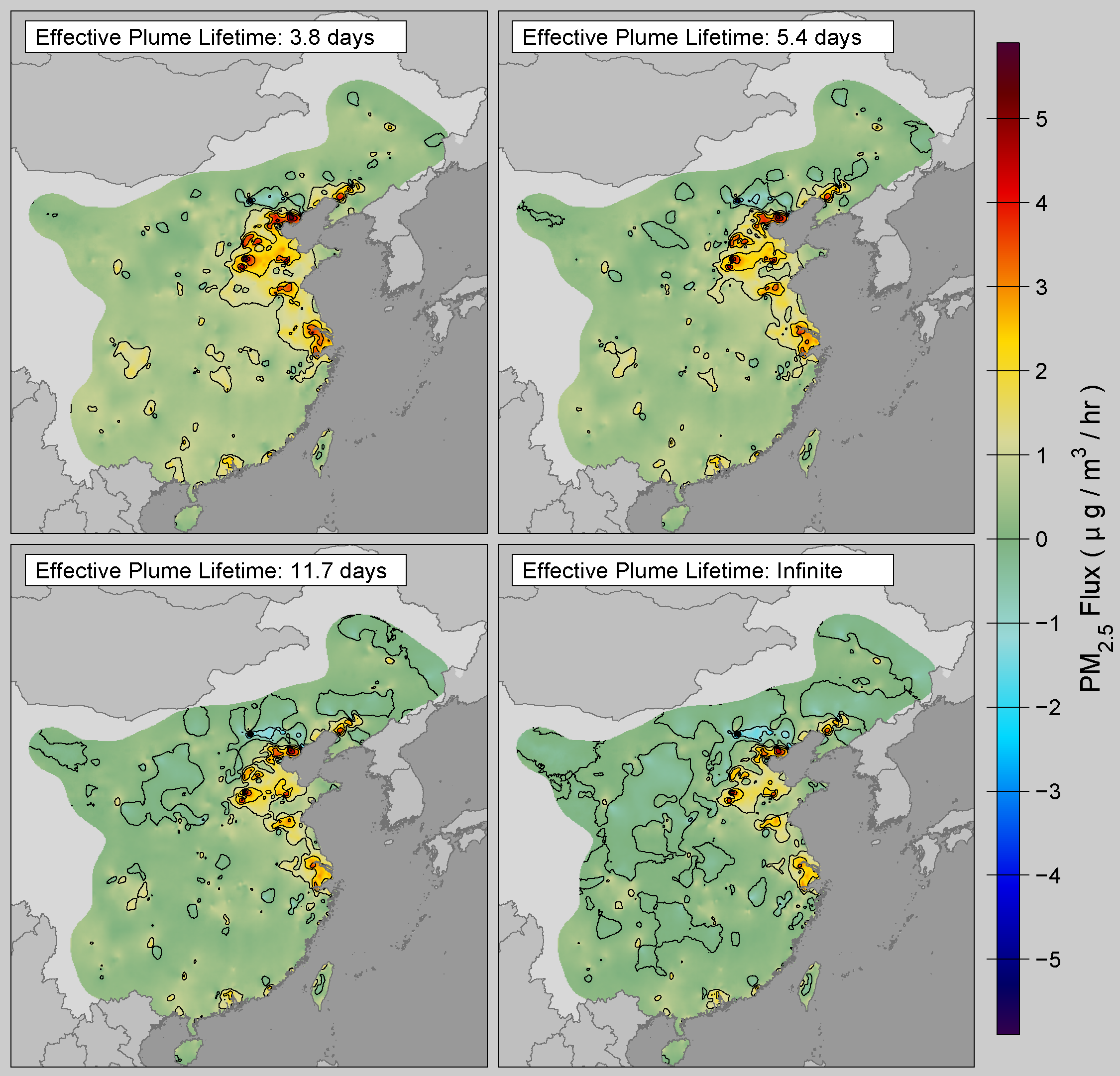

Supplement: S5 Fig — Maps comparing PM2.5 concentrations (top) and fluxes (bottom) during the first half of the sampling period (left column) and the second half of the sampling period (right). PM2.5 concentrations are presented using the same health impact associated color scheme as Fig 3. (TIF) [file pone.0135749.s009.tif]

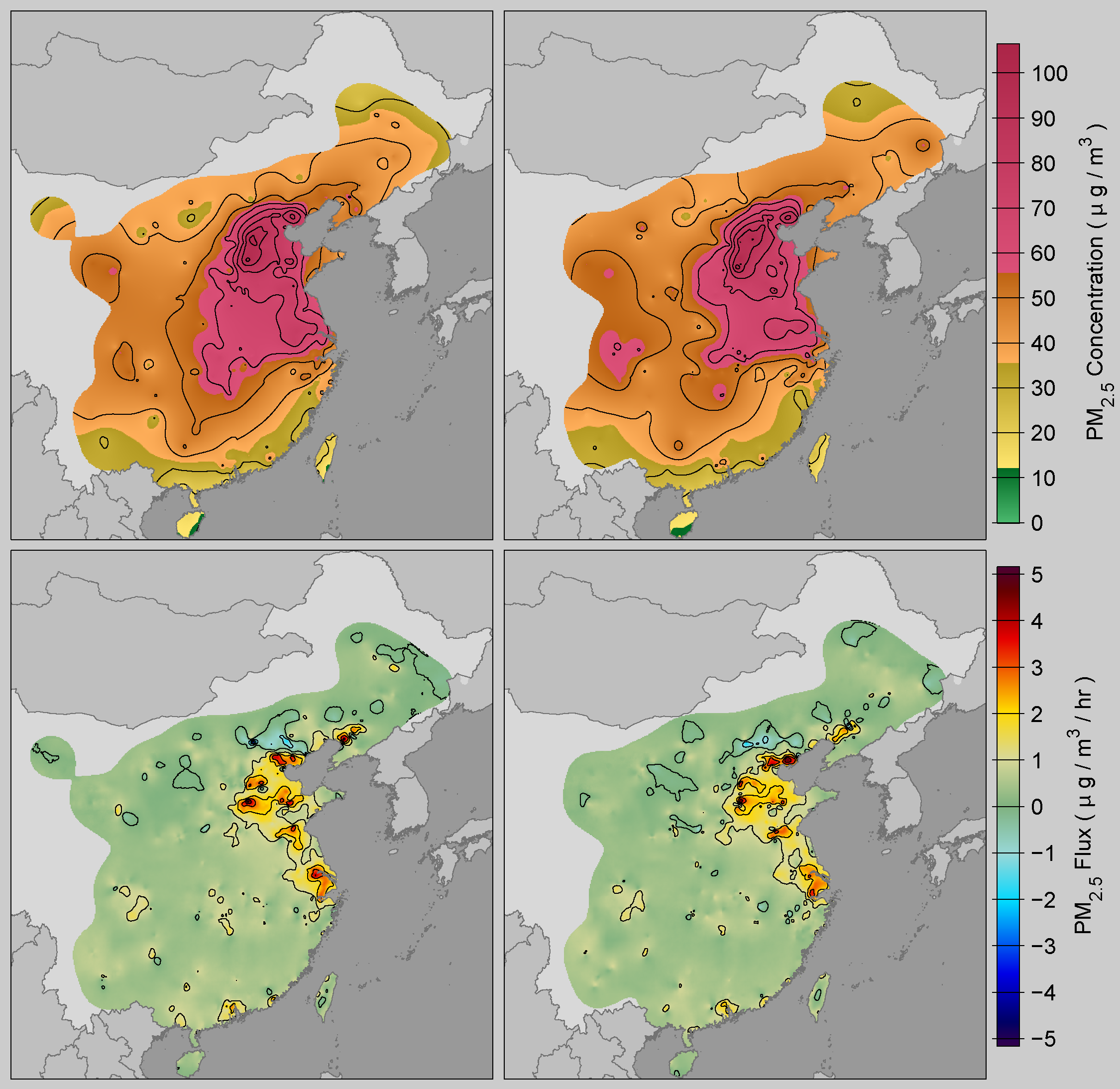

Supplement: S6 Fig — The collection of available stations was randomly assigned to two groups, and the analyses for PM2.5 concentrations (top) and fluxes (bottom) were repeated in full for both groups. These independent subsamples result in similar concentration and flux patterns, implying the reconstructions are likely to be stable with respect to data selection. (TIF) [file pone.0135749.s010.tif]
